# Resveratrol Mitigates Hippocampal Tau Acetylation and Cognitive Deficit by Activation SIRT1 in Aged Rats following Anesthesia and Surgery

**DOI:** 10.1155/2020/4635163

**Published:** 2020-12-16

**Authors:** Jing Yan, Ailin Luo, Rao Sun, Xiaole Tang, Yilin Zhao, Jie Zhang, Biyun Zhou, Hua Zheng, Honghui Yu, Shiyong Li

**Affiliations:** Department of Anesthesiology, Tongji Hospital, Tongji Medical College, Huazhong University of Science and Technology, 1095 Jiefang Avenue, Wuhan, 430030 Hubei, China

## Abstract

Postoperative cognitive dysfunction (POCD) is a sever postsurgical neurological complication in the elderly population. As the global acceleration of population ageing, POCD is proved to be a great challenge to the present labor market and healthcare system. In the present study, our findings showed that tau acetylation mediated by SIRT1 deficiency resulted in tau hyperphosphorylation in the hippocampus of the aged POCD model and consequently contributed to cognitive impairment. Interestingly, pretreatment with resveratrol almost restored the expression of SIRT1, reduced the levels of acetylated tau and hyperphosphorylated tau in the hippocampus, and improved the cognitive performance in the behavioral tests. What is more, we observed that microglia-derived neuroinflammation resulting from SIRT1 inhibition in microglia probably aggravated the tau acetylation in cultured neurons in vitro. Our findings supported the notion that activation SIRT1 provided dually beneficial effect in the aged POCD model. Taken together, our findings provided the initial evidence that tau acetylation was associated with cognitive impairment in the aged POCD model and paved a promising avenue to prevent POCD by inhibiting tau acetylation in a SIRT1-dependent manner.

## 1. Introduction

Postoperative cognitive dysfunction (POCD) is a postsurgical neurological sequela characterized by a reduction in memory, learning, attention, and executive function in the short- and long-term following anesthesia and surgical procedure [1–3]. It is an age-related complication and occurs highly in the geriatric surgical individuals [4]. Clinical findings suggested that this cognitive impairment was associated with prolonged hospitalization, increased mortality, and growing burden of the medical care system [5]. Although a plethora of studies major in elucidating pathophysiologic processes of POCD and exploring feasible targets for therapeutic or preventive intervention, up to date, the neuropathogenesis of POCD remains largely ambiguous.

A growing body of studies showed that Alzheimer's disease- (AD-) like neuropathogenesis was involved in POCD, particularly in the elderly population [6–9]. Tau hyperphosphorylation was extensively investigated and proposed to participate in cognitive decline after anesthesia and surgery [6, 7]. This hypothesis was supported by clinical findings that the classical markers of AD in cerebrospinal fluid(CSF) were associated with postoperative cognitive changes in the elderly surgical population [10–15]. Correspondingly, the abnormal level of specific hyperphosphorylated sites of tau was found in the experimental POCD models [6–8, 15–17]. Concerning the role of tauopathies in neurodegenerative diseases, hyperphosphorylation is one type of neurotoxic modification. Other pathological modifications of tau include acetylation, glycosylation, glycation, acetylation, truncation, and nitration [18, 19]. Among these, tau acetylation was found in the physiological aging and preceded tau hyperphosphorylation in pathological settings [20–24]. A line of studies established that neuronal tau acetylation was directly mediated by SIRT1 and further promoted accumulation of tau hyperphosphorylation by impeding its degradation [21]. Interestingly, our previous and other studies found that the expression of SIRT1 declined in hippocampal microglia with aging and contributed to age-related neuroinflammation and cognitive impairment in rodent [25]. Taken together, these findings raised the possibility that the hippocampal SIRT1 reduction induced tau acetylation and consequently contributed to cognitive decline following anesthesia and surgery.

Resveratrol (3,4,5-trihydroxystilbene) is a natural polyphenol present in various berries, nuts, grapes, and other plants sources [26]. To date, resveratrol has gained growing interest in the neuroscience community due to its pleiotropic neuroprotective effect, such as antioxidative, anti-inflammatory, and antiaging [27]. Furthermore, resveratrol afforded neuroprotection in multiple pathways dependent on activating SIRT1 in neurodegenerative diseases [28–31]. As mentioned before, resveratrol pretreatment activated SIRT1 and mitigated neuroinflammation and cognitive impairment in POCD models in our previous study [25], but it remains unclear whether resveratrol would mitigate surgery and anesthesia-induced SIRT1 reduction and tau acetylation in aged POCD models.

In the present study, we sought to investigate the role of tau acetylation in cognitive decline following surgery and anesthesia in aged rats. Furthermore, we examined the idea that resveratrol would improve the cognitive ability by activating hippocampal SIRT1 and sequentially reducing tau acetylation in aged POCD models.

## 2. Materials and Methods

### 2.1. Animals

**Figure 1:**
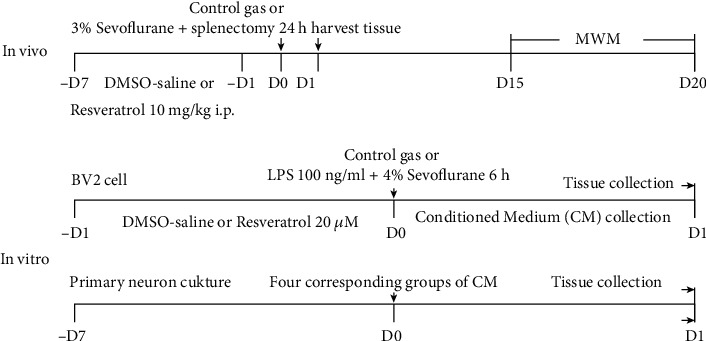
Experimental protocol in vivo and in vitro. In vivo 21-month-old rats were pretreated with DMSO-saline solution or resveratrol via intraperitoneal injection for 7 consecutive days and received control gas or splenectomy under 3% sevoflurane for 2 h. After exposure, part of the rats was decapitated, and tissue samples were collected for biochemistry study, and part of the rats received the MWM test. In vitro BV2 cell was cultured and treated with DMSO-saline solution or resveratrol for 24 h, followed by treatment of control gas or LPS and 4% sevoflurane for 6 h. After exposure, conditioned medium were collected for primary neuron study, and tissue was collected for biochemistry study. After 7-day culture, primary neuron was incubated with four corresponding groups of conditioned medium for 24 h. Tissue was collected for biochemistry study after exposure.

All experimental protocols and animal handling procedures were performed in accordance with the National Institute of Health guidelines and regulations. The experimental protocols were approved by the Committee of Experimental Animals of Tongji Medical College. 21-month-old male Sprague-Dawley rats (weighing 620–670 g at the start of the experiment) were provided by the Center of Experimental Animal of Tongji Medical College. Three to four animals were housed per cage under standard laboratory conditions. As shown in Figure 1, the 21-month-old male rats were randomly assigned to four groups: (1) control+DMSO, rats in the control group were injected with the DMSO-saline solution and treated with a 1 : 1 mix of air and 100% oxygen; (2) sevoflurane+surgery, rats in this group received the DMSO-saline solution followed by splenectomy under sevoflurane anesthesia; (3) resveratrol + DMSO, rats in this group were injected with resveratrol and treated with a 1 : 1 mix of air and 100% oxygen; and (4) sevoflurane+surgery+resveratrol, rats in this group were injected with resveratrol followed by splenectomy under sevoflurane anesthesia.

### 2.2. Anesthesia, Surgery, and Treatment

The anesthesia, surgery, and treatment of each group were shown in Figure 1. The procedure of splenectomy was performed as previously described [25]. Briefly, a small incision approximately 2–3 cm was made in the upper left quadrant, and then the spleen was visualized, isolated, and removed. The surgical procedure was performed under 3% sevoflurane in a 1 : 1 mix of air and 100% oxygen. The wound was infiltrated with 0.25% bupivacaine after the surgery. Rats in the control group were exposed to a 1 : 1 mix of air and 100% oxygen for 2 hours. Resveratrol (Selleck Chemicals, Houston, TX, USA) was prepared by dissolving the powder in a dimethyl sulfoxide- (DMSO-) (Sigma-Aldrich, St. Louis, USA) saline solution and was injected intraperitoneally at 10 mg/kg/day for 7 consecutive days, with the last dose administered 12 hours before surgery. The resveratrol dose was chosen based on a previous study [32].

### 2.3. Cell Lines

The mouse BV2 cell lines were used as an alternative to investigate microglia in vitro. The cell populations were allocated into four groups: (1) control+DMSO, cells in the control group were treated with DMSO-saline solution; (2) LPS+sevoflurane, cells in this group were treated with DMSO+LPS (100 ng/ml, Sigma-Aldrich, St. Louis, USA)+sevoflurane (4% for 6 hours); (3) resveratrol+DMSO, cells in the resveratrol group were treated with resveratrol (20 *μ*mol, Selleck Chemicals); and (4) LPS+sevoflurane+resveratrol, cells in this group were treated with resveratrol (20 *μ*mol)+LPS (100 ng/ml)+sevoflurane (4% for 6 hours). Then, another BV2 cell populations were allocated into four groups (Supplementary Figure S1): (1) control+resveratrol, cells in the control group were treated with resveratrol (20 *μ*mol, Selleck Chemicals); (2) LPS+sevoflurane+resveratrol, cells in this group were treated with DMSO+LPS (100 ng/ml, Sigma-Aldrich, St. Louis, USA)+sevoflurane (4% for 6 hours)+resveratrol (20 *μ*mol); (3) resveratrol+EX527, cells in this group were treated with resveratrol (20 *μ*mol) + EX527 (100 nM, Selleck Chemicals); and (4) LPS+sevoflurane+resveratrol+EX527, cells in this group were treated with resveratrol (20 *μ*mol)+EX527 (100 nM)+LPS (100 ng/ml)+sevoflurane (4% for 6 hours).

### 2.4. Conditioned Medium (CM)

CMs were prepared as described [33]. Briefly, BV2 cells were cultured with DMED-F12 FBS medium overnight and then treated with or without LPS and sevoflurane stimulation in the absence or presence of resveratrol. CM was harvested, centrifuged, filtered, and stored at -80°C until use.

### 2.5. Primary Hippocampal Neurons

Primary hippocampal neurons were prepared from both of the hemispheres hippocampus of postnatal day 1 rat as described [34]. Briefly, rats were anesthetized by CO_2_ and sacrificed, and the hippocampus was isolated and digested in 0.125% trypsin at 37°C for 15-20 min. After centrifuge, cells were cultured with neurobasal medium supplemented with 2% B27, 1% glutamine, and 1% penicillin-streptomycin at 37°C in humidified atmosphere with 5% CO_2_. The medium was changed every 3 days. Neurons cultured for 7 days were used in the study. Neurons were assigned into four groups: (1) control CM, (2) LPS+sevoflurane CM, (3) resveratrol+DMSO CM, and (4) LPS + sevoflurane+resveratrol CM and treated with corresponding CM for 24 hours. Then, another primary neurons were assigned into four groups: (1) control+resveratrol CM, (2) LPS+sevoflurane+resveratrol CM, (3) resveratrol+EX527 CM, and (4) LPS+sevoflurane+resveratrol+EX527 CM and treated with corresponding CM for 24 hours.

### 2.6. Western Blot Analysis

Western blot was performed as previously reported [35]. The hippocampal tissue was harvested 24 hours after anesthesia and surgery and homogenized at 4°C for 30 min in RIPA lysis buffer. Cells were washed with PBS buffer three times 24 hours after treatment and collected into clean centrifuge tubes. The protein levels in the hippocampal tissues and cells were determined by a BCA assay kit (Boster, Wuhan, China). An equal amount of protein was loaded and separated by SDS-PAGE and transferred to a polyvinylidene difluoride (PVDF) membrane (Millipore, Bedford, MA, USA) by electrophoresis. The membranes were blocked with 5% nonfat skim milk in TBST (0.1% Tween 20 in TBS) for 45 min at room temperature and then incubated overnight at 4°C with an anti-SIRT1 (1 : 1000; Abcam, Cambridge, UK), anti-ac-tau (k280) (1 : 1000, Anaspec, CA, USA), anti-ac-tau (k686) (1 : 1000, EnoGene, Nanjing, China), anti-p-tau (AT8) (1 : 1000, Thermo Fisher Scientific, MA, USA), anti-Acetyl-NF-kappaB (1 : 1000, Affinity, OH, USA), anti-total tau (1 : 1000, Affinity), or anti-*β*-actin (1 : 1000, Affinity) antibody. On the second day, the membranes were washed three times with PBST and then incubated with a horseradish peroxidase- (HRP-) conjugated goat anti-mouse or goat anti-rabbit IgG antibody (1 : 2000; Abbkine, Wuhan, China) for 2 hours at room temperature. Bands were quantified using laboratory imaging software, and the experiments were repeated in triplicate.

### 2.7. Immunofluorescence

Immunofluorescence staining was performed as described [25, 36]. Briefly, rats were sacrificed with sodium pentobarbital (85 mg/kg) 24 hours after anesthesia and surgery. Brains were harvested, fixed, and dehydrated. Ten-micron-thick frozen hippocampal sections were cut and incubated with 5% normal donkey serum in PBS for 1 hour, followed by incubation with an anti-SIRT1 (1 : 1000; Abcam) and anti-NeuN (1 : 1000; Millipore, USA) antibody at 4°C overnight. Sections were incubated with donkey anti-Goat IgG conjugated to Alexa Fluor®594 (1 : 500, Abbkine) and donkey anti-Mouse IgG conjugated to Alexa Fluor®488 (1 : 500, Abbkine) in the dark for 1.5 hour at room temperature. Images were acquired with a fluorescence microscope.

### 2.8. Morris Water Maze Test

The Morris water maze test was performed on days 15-20 after anesthesia and surgery as previously described [37]. Briefly, the training protocol for the task of the MWM test consisted of 3 trials (120 s maximum; interval 20 min) each day for five consecutive days. The probe trial (120 s), in which the platform was removed, was performed 24 hours after the end of the fifth day training.

### 2.9. Statistical Analysis

The data obtained from biochemistry studies and escape latency of MWM were represented as the mean ± SD. The data of platform crossing time of MWM were presented as medians with interquartile range. The data were analysed with GraphPad Prism 7.0 (GraphPad Software, CA, USA). Statistical evaluation between 2 groups was performed using two-sided Student's *t* test. Statistical evaluation between 4 groups in biochemistry studies was performed using two-way analysis of variance (ANOVA) without repeated measurement to evaluate the interaction of group and treatment. Data of escape latency in the MWM test were measured by two-way ANOVA with repeated measurement, followed by the Bonferroni posthoc. The Mann–Whitney *U* test was used to compare the difference in the platform-crossing times in the MWM test. *P* < 0.05 was considered statistically significant.

## 3. Results

### 3.1. Anesthesia and Surgery Elevates Tau Acetylation and Decreases the Neuronal SIRT1 Expression in the Hippocampus of Aged Rats

**Figure 2:**
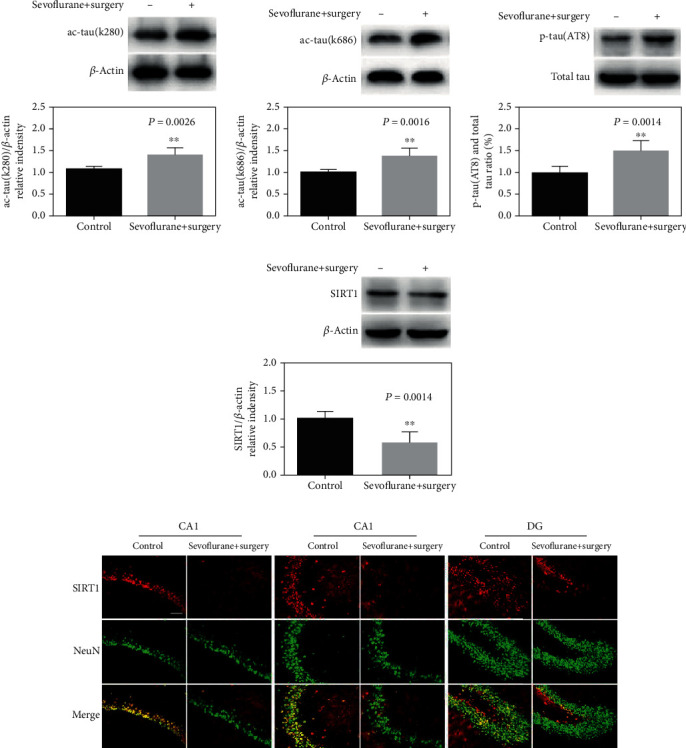
Anesthesia and surgery elevate tau acetylation and decrease the neuronal SIRT1 expression in the hippocampus of aged rats. (a)–(d) Representative immunoblot bands and the corresponding densitometry analysis of ac-tau (k280), ac-tau (k686), SIRT1 expression normalized to *β*-actin, and p-tau (AT8) normalized to total tau. (e) Immunostaining of SIRT1 (red) and NeuN (green) in the control and sevoflurane+surgery groups. Data are presented as the mean ± SD. *n* = 6 per group. Scale bar = 50 *μ*m.

First, we investigated tau acetylation levels after anesthesia and surgery of aged rats. The data showed that the expression of ac-tau (k280) and ac-tau (k686) was significantly increased in the hippocampus after anesthesia and surgery (*P* = 0.0026; *P* = 0.0016, Figures 2(a) and 2(b)). Tau acetylation can precede tau hyperphosphorylation in the pathological context, and we further elevated the expression of tau phosphorylation levels. As shown in Figure 1(c), anesthesia and surgery led to a significant increase in the p-tau (AT8) expression in the hippocampus (*P* = 0.0014). Similar to the result with our previous study, anesthesia and surgery significantly decreased the expression of SIRT1 in the hippocampus (*P* = 0.0014, Figure 2(d)). Interestingly, the immunofluorescence results revealed that neuronal SIRT1 was notably decreased in CA1, CA3, and DG regions of the hippocampus after anesthesia and surgery (Figure 2(e)).

### 3.2. Resveratrol Pretreatment Increases the Neuronal SIRT1 Expression in the Hippocampus of Aged Rats Exposed to Anesthesia and Surgery

**Figure 3:**
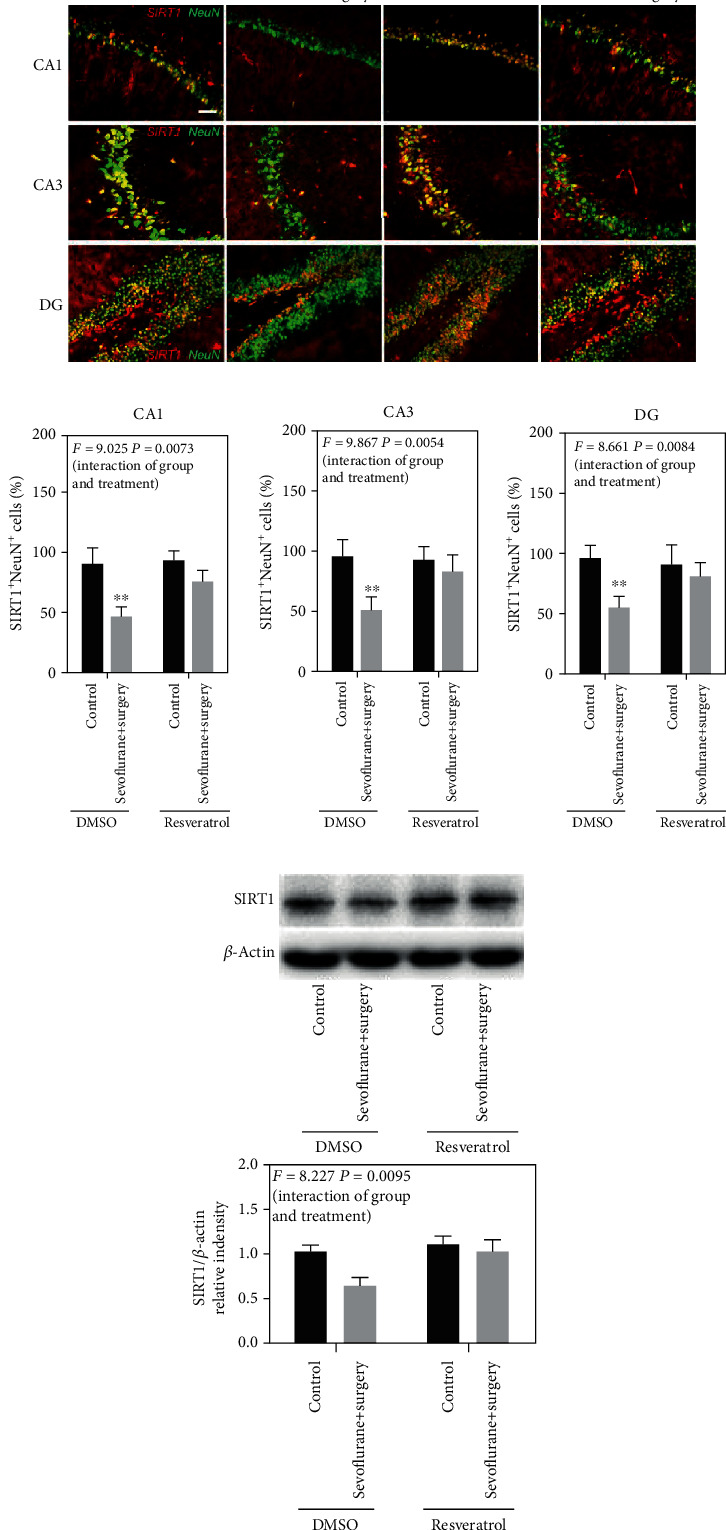
Resveratrol pretreatment increases the neuronal SIRT1 expression in the hippocampus of aged rats exposed to anesthesia and surgery. (a) Immunostaining of SIRT1 (red) and NeuN (green). (b)–(d) Quantification of SIRT1-positive and NeuN-positive cells. (e) Representative immunoblot band and the corresponding densitometry analysis of the SIRT1 expression normalized to *β*-actin. Data are presented as the mean ± SD. *n* = 6 per group. Scale bar = 50 *μ*m.

To verify the function of resveratrol on the SIRT1 expression, we used both immunostaining and western blot to detect SIRT1 in the hippocampus. As shown in Figure 3(a), there is a clear trend of decreasing neuronal SIRT1 levels in CA1, CA3, and DG regions of the hippocampus in the sevoflurane+surgery group compared with the control group, and resveratrol pretreatment blocked the sevoflurane and surgery-induced changes in neuronal SIRT1 levels (*F* = 9.025, *P* = 0.0073; *F* = 9.867, *P* = 0.0054; *F* = 8.661, *P* = 0.0084, Figures 3(b)–3(d)). The western blot data also declared that resveratrol pretreatment remarkably blocked sevoflurane and surgery-induced reduction in SIRT1 levels in the hippocampus (*F* = 8.227, *P* = 0.0095, Figure 3(e)).

### 3.3. Activation of SIRT1 Decreases Tau Acetylation and Tau Phosphorylation in the Hippocampus of Aged Rats Exposed to Anesthesia and Surgery

**Figure 4:**
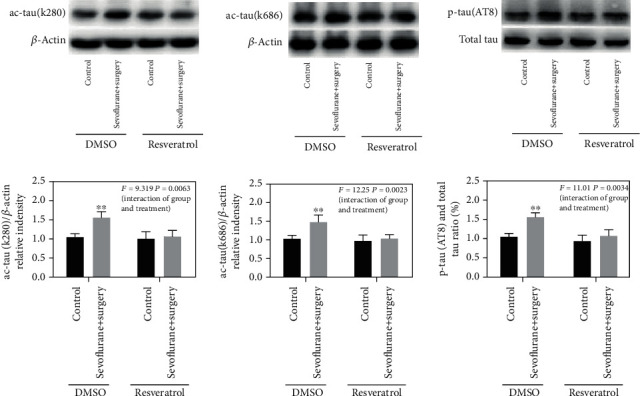
Activation of SIRT1 decreases tau acetylation and tau phosphorylation in the hippocampus of aged rats exposed to anesthesia and surgery. (a)–(c) Representative immunoblot bands and the corresponding densitometry analysis of ac-tau (k280), ac-tau (k686) expression normalized to *β*-actin, and p-tau (AT8) expression normalized to total tau. Data are presented as the mean ± SD. *n* = 6 per group.

To verify the function of SIRT1 in the mediation of tau acetylation and tau phosphorylation in the aged POCD model, resveratrol was used to activate the expression of SIRT1. The data showed that activation of SIRT1 reduced anesthesia and surgery-induced tau acetylation and tau phosphorylation, as showed by the increased levels of ac-tau (k280), ac-tau (k686), and p-tau (AT8) in the sevolurane+surgery group compared with the control group, and decreased levels of ac-tau (k280), ac-tau (k686), and p-tau (AT8) in the sevoflurane+surgery+resveratrol group (*F* = 9.319, *P* = 0.0063; *F* = 12.25, *P* = 0.0023; *F* = 11.01, *P* = 0.0034, Figures 4(a)–4(c)).

### 3.4. Activation of SIRT1 Ameliorates Cognitive Impairment in Aged Rats of the POCD Model

**Figure 5:**
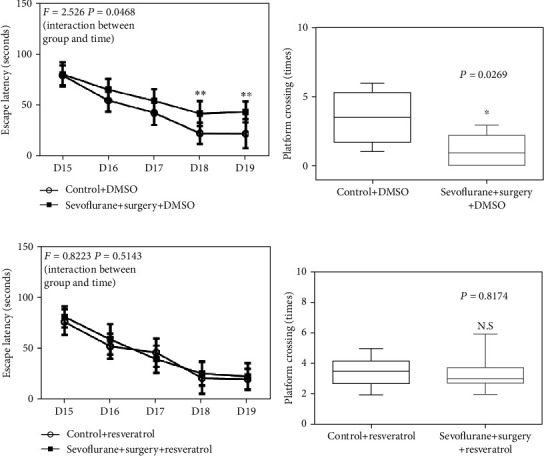
Activation of SIRT1 ameliorates cognitive impairment in aged rats of the POCD model. (a) The difference of escape latency in the MWM in five training days between control and sevoflurane+surgery groups. (b) The difference of platform crossing times in the probe trial of the MWM between control and sevoflurane+surgery groups. (c) The difference of escape latency in the MWM in five training days between resveratrol and sevoflurane+surgery+resveratrol groups. (d) The difference of platform crossing times in the probe trial of the MWM between resveratrol and sevoflurane+surgery+resveratrol groups. Data are presented as the mean ± SD. *n* = 10 per group.

To explore the relationship between the SIRT1 level in the hippocampus and cognitive decline in aged rats of the POCD model, resveratrol was used to activate the expression of SIRT1 in the hippocampus, and Morris water maze was used to evaluate the spatial learning and memory performance of aged rats. During the training phase of MWM, anesthesia and surgery induced cognitive impairment as evidenced by the significant interaction between treatment (control+DMSO condition and sevoflurane+surgery) and time on escape latency (*F* = 2.526, *P* = 0.0468, Figure 5(a)). A Bonferroni posthoc test showed that rats in the sevoflurane+surgery group spent more time locating the hidden platform on the fourth and fifth training day compared with rats in the control group (*P* < 0.01, Figure 5(a)). In the probe trial, the rats in the sevoflurane+surgery group showed a remarkable reduction in the platform crossing times (*P* = 0.0269, Figure 5(b)). Importantly, the cognitive impairment was prevented by resveratrol pretreatment, as shown by no significant interaction between treatment (control+resveratrol condition and sevoflurane+surgery+resveratrol) and time on escape latency (*F* = 0.8223, *P* = 0.5143, Figure 5(c)), and no significant difference in platform crossing times between the control+resveratrol group and sevoflurane+surgery+resveratrol group (*P* = 0.8174, Figure 5(d)).

### 3.5. Activation of SIRT1 Reduces LPS+Sevoflurane-Induced Ac-NF-*κ*B and Proinflammatory Cytokine Expression in the BV2 Cell Line

**Figure 6:**
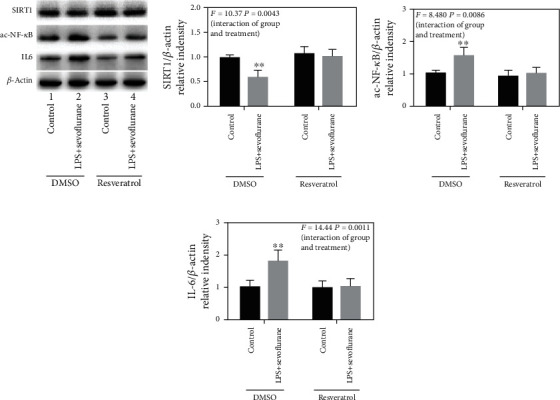
Activation of SIRT1 reduces the LPS + sevoflurane-induced ac-NF-*κ*B and proinflammatory cytokine expression in BV2 cell lines. (a) Representative immunoblot bands of SIRT1, ac-NF-*κ*B, and IL-6 expression in BV2 cell lines. (b)–(d) The corresponding densitometry analysis of SIRT1, ac-NF-*κ*B, and IL-6 expression normalized to *β*-actin. Data are presented as the mean ± SD. *n* = 6 per group.

Our previous study had found that SIRT1 mediated neuroinflammation via regulation of ac-NF-*κ*B in the hippocampus of an aged POCD model. To confirm the relationship of SIRT1 and neuroinflammation, LPS and sevoflurane were used as stimulus to induce the proinflammatory cytokine expression in BV2 cell lines. As showed in Figures 6(a)–6(d), LPS and sevoflurane led to a notable decrease in the SIRT1 expression and increase in the levels of ac-NF-*κ*B and IL-6 in BV2 cell lines. However, resveratrol treatment resulted in a remarkable increase in the SIRT1 expression and decrease in the levels of ac-NF-*κ*B and IL-6 compared with that in BV2 cell lines in the LPS+ sevoflurane group (*F* = 10.37, *P* = 0.0043; *F* = 8.480, *P* = 0.0086; *F* = 14.44, *P* = 0.0011, Figures 6(b)–6(d)). As resveratrol is a polyphenol with pleiotropic neuroprotective properties, we explored EX527, a SIRT1 inhibitor, to validate our hypothesis. We found EX527 obviously weakened the effect of resveratrol which decreased the level of SIRT1 and increased the levels of ac-NF-*κ*B and IL-6 in BV2 cell lines (as shown in Figure S2. A-D). These findings suggested that resveratrol reduced LPS+sevoflurane-induced neuroinflammation by, at least partially, acting on SIRT1 in the BV2 cell line.

### 3.6. Activation of SIRT1 Decreased the LPS+Sevoflurane-Conditioned Medium-Induced Tau Acetylation and Tau Phosphorylation in Primary Hippocampal Neurons

**Figure 7:**
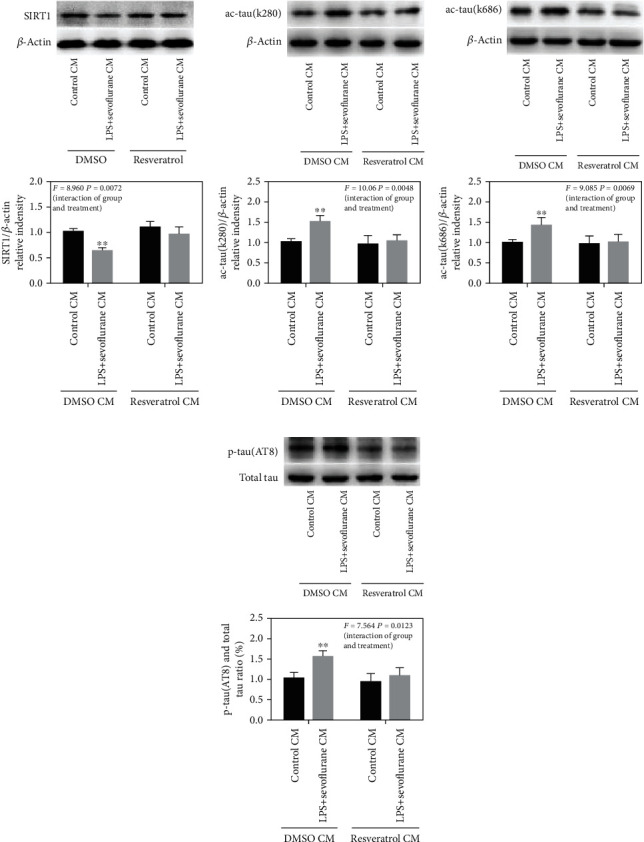
Activation of SIRT1 decreased the LPS+sevoflurane-conditioned medium-induced tau acetylation and tau phosphorylation in primary hippocampal neurons. (a)–(d) Representative immunoblot bands and the corresponding densitometry analysis of SIRT1, ac-tau (k280), ac-tau (k686) expression normalized to *β*-actin, and p-tau (AT8) expression normalized to total tau. Data are presented as the mean ± SD. *n* = 6 per group.

To explore the underline mechanism of neuroinflammation-induced tau acetylation in aged POCD models, conditioned medium of BV2 cells was collected to treat in primary hippocampal neurons. The results indicated that LPS+sevoflurane CM led to a decrease in the SIRT1 expression in primary hippocampal neurons compared with the control CM group, and resveratrol involved conditioned medium induced a remarkable increase in the SIRT1 level (*F* = 8.960, *P* = 0.0072, Figure 7(a)). Besides, LPS+sevoflurane CM induced tau acetylation and tau phosphorylation in primary hippocampal neurons, as shown by increased levels of ac-tau (k280), ac-tau (k686) ,and p-tau (AT8) compared with those in primary hippocampal neurons in the control CM group, whereas resveratrol CM treatment showed remarkable downregulation in ac-tau (k280), ac-tau (k686), and p-tau (AT8) expression (*F* = 10.06, *P* = 0.0048; *F* = 9.085, *P* = 0.0069; *F* = 7.564, *P* = 0.0123, Figures 7(b)–7(d)). However, SIRT1 inhibitor EX527 weakened the effect of resveratrol on tau acetylation and tau phosphorylation by reducing the SIRT1 expression (as shown in Figure S3. A-D). Taken together, SIRT1 activation was associated with resveratrol's effect on LPS+sevoflurane-conditioned medium-induced tau acetylation and tau phosphorylation.

## 4. Discussion

In the present study, we showed that elevated acetylation of tau was mediated by SIRT1 reduction following surgery and anesthesia in aged rat of the POCD model. Pretreatment with resveratrol, a natural activator of SIRT1, mitigated SIRT1 suppression, further downregulated the level of tau acetylation and hyperphosphorylation in the hippocampus and consequently mitigated the cognitive decline of the aged POCD model. Furthermore, we found that microglial cell line-derived neuroinflammation was associated with neuronal tau acetylation in the conditioned culture system in vitro. These findings suggest that tau acetylation is at the nexus of transient neuroinflammation to the prolonged cognitive decline following surgery and anesthesia in the aged POCD models.

POCD is a severe neurological complication, and the symptoms last for days to months or years [3, 38]. Accumulating of well-documented studies regarded neuroinflammation as the main pathological culprit in the POCD model [39–43]. As neuroinflammation is usually a transient process, tau hyperphosphorylation was once hypothesized as the bridge between neuroinflammation and long-term cognitive impairment in POCD [9]. This idea was supported by previous reports that hyperphosphorylated tau resulted in abnormality of axonal transport, synaptic structure and function, and finally induced cognitive impairment in AD [19, 44, 45]. Several studies found tau hyperphosphorylated in the preexisted AD model following surgery and anesthesia [7, 46–48]. As known, tauopathies include a wide range of abnormal modifications of tau in neurodegenerative diseases [18, 19]. However, it remains poorly understood whether other forms of tauopathies are involved in POCD.

Acetylation is one type of posttranslational modifications of tau [21]. And then a growing number of findings have linked the acetylation of tau to tau neurotoxicity [20–24, 49, 50]. The initial evidence for tau acetylation and its role in AD turned up in 2010 [21]. It showed that tau hyperacetylation was an early event in AD and occurred before the accumulation of tau tangles [20–22, 24]. After this report, tau acetylation was observed in various neurodegenerative diseases. Hyperacetylated tau underlay the neuropathogenesis in a variety of manners, including preventing tau degradation, promoting tau aggregation and propagation, and disrupting synaptic structure and function [20–22, 24, 49, 50]. In the present study, we found that tau acetylation was elevated in the hippocampi of aged rats following anesthesia and surgery. To the best of our knowledge, this is the initial evidence that shows that tau acetylation is associated with cognitive impairment in POCD models. This finding provides insight into exploring the mechanism of POCD and possibly opens a new acetylated tau-targeted therapeutics pipeline for POCD.

Tau acetylation is mediated by acetyltransferases p300 or deacetylases SIRT1 in the pathological condition [21]. SIRT1 is the NAD-dependent class III deacetylase and strongly implicated in various pathophysiological process, including neurodevelopment, aging, stress, inflammation, and cancer [51, 52]. In terms of modulating tau acetylation, SIRT1 could interact with tau directly. SIRT1 reduction led to increased tau acetylation and elevated level of tau phosphorylation, while restoring the SIRT1 expression reduced the level of acetylated and phosphorylated tau [21]. In line with these findings, our present and previous studies observed hippocampal SIRT1 reduction in aged rats of the POCD model [25]. Remarkably, pretreating the model with resveratrol nearly restored the hippocampal expression of SIRT1, suppressed the levels of tau acetylation and phosphorylation, and finally mitigated the cognitive impairment. Base on the present study, we did not rule out the role of p300 that was in tau acetylation in the POCD model and set out to conduct further study to examine this possibility. In summary, our finding suggested that SIRT1 reduction contributed to tau acetylation and cognitive impairment in aged rats following surgery and anesthesia.

Neuroinflammation was found to be involved in tau phosphorylation in AD and POCD. In fact, neuroinflammation was considered as the mainstream neuropathogenesis of POCD in preclinical studies [39–43, 53–57]. In the present study, we sought to examine whether it was a link between neuroinflammation and tau acetylation in aged POCD models. It was reported previously that decrease of microglial SIRT1 contributed to aging-related neurodegeneration and cognitive decline [58, 59]. Our previous study found that microglial-derived SIRT1 reduction contributed to neuroinflammation and cognitive impairment in the aged POCD model [25]. However, SIRT1-mediated tau acetylation was found in neurons [21]. As SIRT1 is found both in neurons and microglia, how might neuroinflammation lead to tau acetylation in the aged POCD model? We hypothesized that overactivated neuroinflammation inhibited neuronal SIRT1 and further induced tau hyperacetylation.

To test this hypothesis, we used the conditioned medium cell cultures. In agreement with previous studies [60], we found that SIRT1 was reduced in microglial cell line (BV2), and neuroinflammation was induced by lipopolysaccharide (LPS)+sevoflurane. Incubating neurons with the conditioned medium from LPS+sevoflurane-stimulated BV2 culture medium led to neuronal reduction of SIRT1 and increase of tau acetylation and tau phosphorylation. Conditioned medium pretreated with resveratrol mitigated neuronal SIRT1 reduction and tau acetylation and tau phosphorylation. However, SIRT1 inhibitor EX527 weakened resveratrol's anti-neuroinflammatory and tau-reducing effect. This finding indirectly suggested that the effect of resveratrol on reducing neuroinflammation and tau acetylation and tau phosphorylation was mediated by SIRT1. SIRT1 is also a redox-sensitive sensor. This idea was supported by findings that oxidative stress inhibited the SIRT1 expression and causally led to pathological changes with LPS stimulus [61]. In this regard, we could not rule out the possibility that reactive oxygen species(ROS) released by BV2 activation contributed to neuronal SIRT1 inhibition, because increased ROS was generated in aged rodents of POCD models and associated with cognitive impairment [62–64]. Interestingly, the cerebrospinal fluid level of 15-F_2t_-isoprostane, a specific biomarker of oxidative stress, is increased with aging and exacerbation of cognitive dysfunction [65, 66]. In this regard, these data open a new direction for diagnosis of POCD in the aged population. Taken together, these data suggested that microglia-derived neuroinflammation reduced the level of SIRT1 in hippocampal neurons in vitro.

Resveratrol (3,4′,5-trihydroxystilbene, C_14_H_12_O_3_) is a natural polyphenolic nutraceutical that can be extracted from grapes, the roots of white hellebore, and *Polygonum cuspidatum* [26]. It exhibits pleiotropic activities, including antioxidation, anti-inflammation, antiapoptosis, anticancer, and antiaging under a wide variety of pathological condition [67, 68]. Although the mechanism is not yet clear by which resveratrol confers such a vast range of beneficial effects across various disease models, a wealth of evidence points its neuroprotective property to the sirtuins family, in particular, the SIRT1 [27, 69]. Resveratrol is not a specific activator of SIRT1, but accumulating findings suggest that resveratrol exerts neuroprotection via activation of SIRT1 in neurodegenerative diseases and stroke [21, 29, 31, 58, 70]. In light of the finding that SIRT1 reduction was observed in conditioned medium of the microglial cell line and conditioned medium treated neurons and the fact SIRT1 directly modulated tau acetylation, resveratrol likely afforded the neuroprotective effect by acting on the dual neuropathological changes neuroinflammation and tau acetylation. Indeed, we found that resveratrol pretreatment inhibited neuroinflammation, reduced the levels of acetylated tau and phosphorylated tau, and mitigated the cognitive decline in the aged rat of the POCD model. Considering the efficacy, safety, and pharmacokinetics of resveratrol that have been documented in more than 200 clinical trials [27], but yet absent in the POCD-related trial, we suppose that resveratrol might be a promising measurement to prevent POCD in the elderly surgical population.

There are several limitations to the present study. First, we did not investigate whether tau acetylation and tau phosphorylation worked in parallel or in succession as previously reported in AD. Second, we did not examine whether other enzymes modulated tau acetylation in the present model. Third, our surgery procedure was splenectomy, but not the prevalent laparotomy or tibial fracture, to keep consistency with our previous model. Further, study would be conducted to overcome the flaws of the current study.

## 5. Conclusions

Collectively, this study provides initial evidence that increased hippocampal tau acetylation which was associated with neuroinflammatory stress in aged rat of the POCD model. The hyperacetylated tau, at least partially mediated by SIRT1 inhibition, increased tau phosphorylation and contributed to cognitive impairment in the present experimental model. However, preconditioning with resveratrol mitigated the tau hyperacetylation and hyperphosphorylation and improved the behavioral performance in the learning and memory test. This study proposed a new neuropathological mechanism mediating cognitive impairment in the aged POCD model and paved a promising way to prevent this postoperative neurological complication.

## Data Availability

The data used to support the findings of this study are included within the article.

## Supplementary Materials

Supplementary Materials Figure S1: experimental protocol in vitro. The BV2 cell was cultured and treated with resveratrol or EX527 for 24 h, followed by treatment of control gas or LPS and 4% sevoflurane for 6 h. After exposure, conditioned medium was collected for primary neuron study, and tissue was collected for biochemistry study. After the 7-day culture, primary neuron was incubated with four corresponding groups of conditioned medium for 24 h. Tissue was collected for biochemistry study after exposure. Figure S2: EX527 weakened the effect of resveratrol in reducing LPS+sevoflurane-induced ac-NF-*κ*B and proinflammatory cytokine expression in BV2 cell lines. (a) Representative immunoblot bands of SIRT1, ac-NF-*κ*B, and IL-6 expression in BV2 cell lines. (b)–(d) The corresponding densitometry analysis of SIRT1, ac-NF-*κ*B, and IL-6 expression normalized to *β*-actin. Data are presented as the mean ± SD. *n* = 6 per group. Figure S3: EX527 weakened the effect of resveratrol in decreasing the LPS+sevoflurane-conditioned medium-induced tau acetylation and tau phosphorylation in primary hippocampal neurons. (a)–(d) Representative immunoblot bands and the corresponding densitometry analysis of SIRT1, ac-tau (k280), ac-tau (k686) expression normalized to *β*-actin, and p-tau (AT8) expression normalized to total tau. Data are presented as the mean ± SD. *n* = 6 per group.
