# Supplementary material for: Resveratrol Mitigates Hippocampal Tau Acetylation and Cognitive Deficit by Activation SIRT1 in Aged Rats following Anesthesia and Surgery
Source: Oxid Med Cell Longev. 2020 Dec 16;2020:4635163. doi: 10.1155/2020/4635163 (PMC7758127; doi:10.1155/2020/4635163)
Supplement: Supplementary Materials — Figure S1: experimental protocol in vitro. The BV2 cell was cultured and treated with resveratrol or EX527 for 24 h, followed by treatment of control gas or LPS and 4% sevoflurane for 6 h. After exposure, conditioned medium was collected for primary neuron study, and tissue was collected for biochemistry study. After the 7-day culture, primary neuron was incubated with four corresponding groups of conditioned medium for 24 h. Tissue was collected for biochemistry study after exposure. Figure S2: EX527 weakened the effect of resveratrol in reducing LPS+sevoflurane-induced ac-NF-κB and proinflammatory cytokine expression in BV2 cell lines. (a) Representative immunoblot bands of SIRT1, ac-NF-κB, and IL-6 expression in BV2 cell lines. (b)–(d) The corresponding densitometry analysis of SIRT1, ac-NF-κB, and IL-6 expression normalized to β-actin. Data are presented as the mean ± SD. n = 6 per group. Figure S3: EX527 weakened the effect of resveratrol in decreasing the LPS+sevoflurane-conditioned medium-induced tau acetylation and tau phosphorylation in primary hippocampal neurons. (a)–(d) Representative immunoblot bands and the corresponding densitometry analysis of SIRT1, ac-tau (k280), ac-tau (k686) expression normalized to β-actin, and p-tau (AT8) expression normalized to total tau. Data are presented as the mean ± SD. n = 6 per group. [file 4635163.f1.docx]

**supplementary figures**





Figure S1. Experimental protocol *in vitro*. BV2 cell was cultured and treated with resveratrol or EX527 for 24 h, followed by treatment of control gas or LPS and 4% sevoflurane for 6 h. After exposure, conditioned medium were collected for primary neuron study, and tissue were collected for biochemistry study. After 7-day culture, primary neuron were incubated with four corresponding groups of conditioned medium for 24 h. Tissue were collected for biochemistry study after exposure.


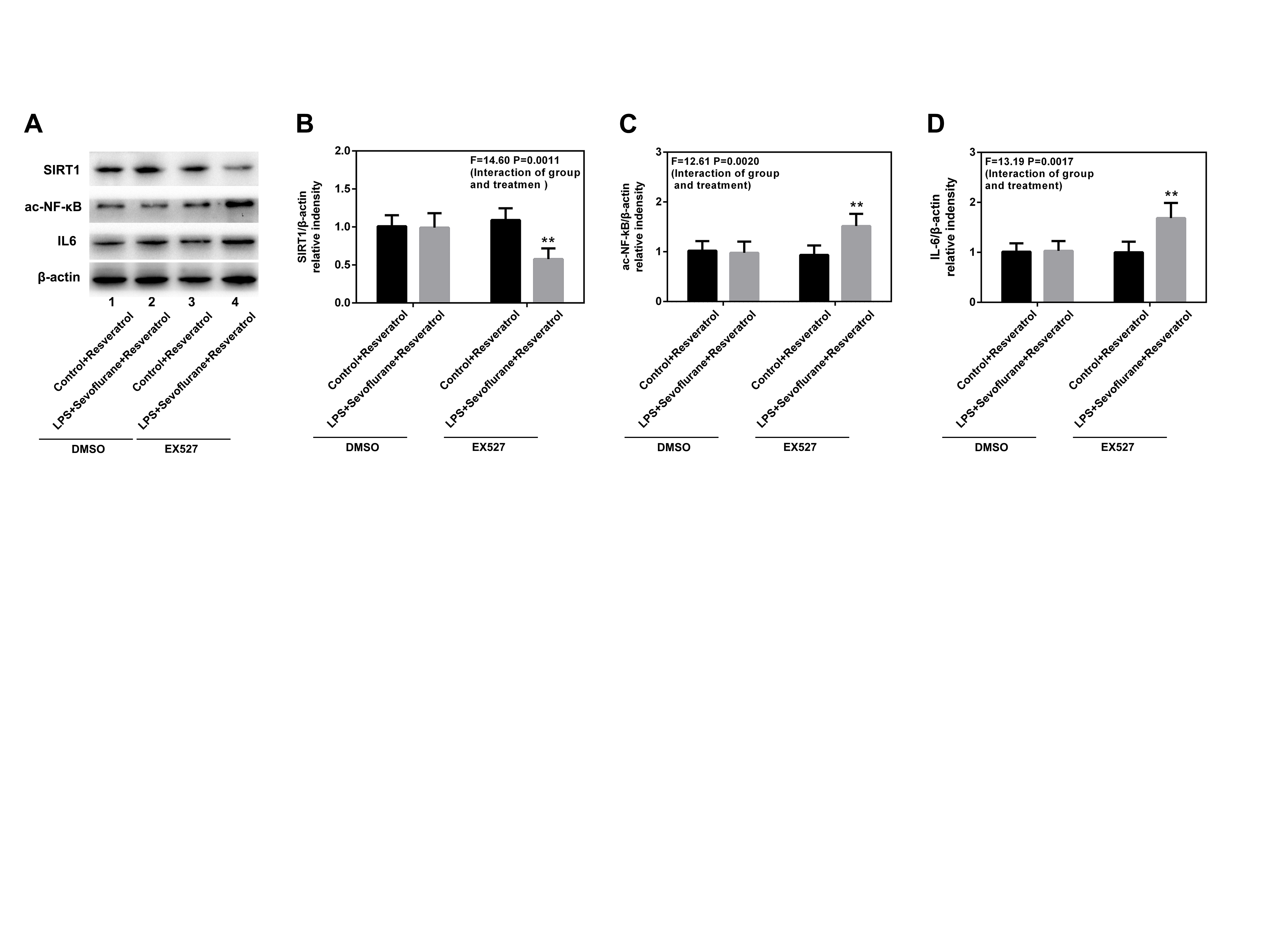


Figure S2. EX527 weakened the effect of resveratrol in reducing LPS+Sevoflurane-induced ac-NF-κB and proinflammatory cytokines expression in BV2 cell lines. (A) Representative immunoblot bands of SIRT1, ac-NF-κB and IL-6 expression in BV2 cell lines. (B-D) The corresponding densitometry analysis of SIRT1, ac-NF-κB and IL-6 expression normalized to β-actin. Data are presented as the mean ± SD. n= 6 per group.


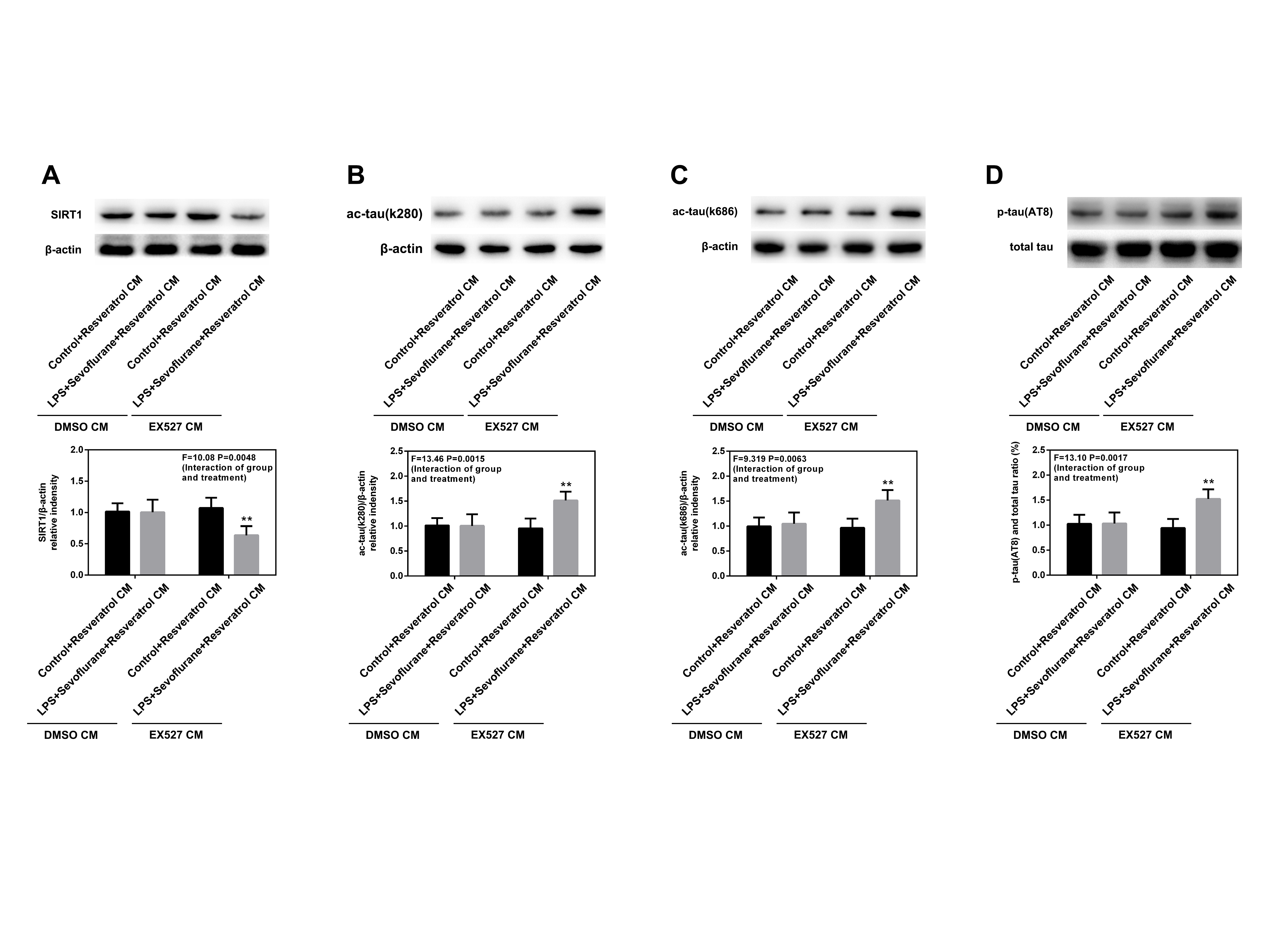


Figure S3. EX527 weakened the effect of resveratrol in decreasing the LPS + Sevoflurane-Conditioned medium-induced tau acetylation and tau phosphorylation in primary hippocampal neurons. (A-D) Representative immunoblot bands and the corresponding densitometry analysis of SIRT1, ac-tau (k280), ac-tau (k686) expression normalized to β-actin, and p-tau (AT8) expression normalized to total tau. Data are presented as the mean ± SD. n= 6 per group.
